# Full Genome Sequence of an Avian Influenza H5N1 Virus Isolated from the Environment in Hunan Province, China

**DOI:** 10.1128/genomeA.00088-13

**Published:** 2013-05-16

**Authors:** Ba Wang, Hongbo Zhang, Quanjiao Chen, Ze Chen

**Affiliations:** College of Life Sciences, Hunan Normal University, Changsha, Hunan, Chinaa;; Center for Emerging Infectious Diseases, Wuhan Institute of Virology, Chinese Academy of Sciences, Wuhan, Hubei, Chinab;; Shanghai Institute of Biological Products, Shanghai, Chinac

## Abstract

We isolated an avian influenza virus A/environment/Hunan/3/2011(H5N1) from a body of water in Hunan, China. The nucleotide sequence of the virus shares 95% homology with H5N1 from the east Asia region. Phylogenetic analysis indicates that its HA gene belongs to clade 2.3.2.1 and that other internal genes present different recombination features.

## GENOME ANNOUNCEMENT

In 1997, it was first demonstrated in Hong Kong that the highly pathogenic H5N1 avian influenza virus (AIV) could endanger human lives (1). Waterfowl around wetlands and lakes have been considered the natural host of AIV (2). Studies so far have shown that multiple AIV subtypes can maintain infectivity in water bodies for months at low temperatures and for over 1 week at 22°C (3).

As water bodies are an important transmission route for AIV, their contamination with AIV could likely cause transmission of AIV between migratory birds and domestic poultry. Therefore, persistent surveillance of AIV in water bodies appears to be particularly important. In March 2011, we isolated a strain of AIV subtype H5N1 from the waters of a wild bird habitat in the Xiangtan area (east longitude 111°58′ to 113°05′, north latitude 27°20′55″ to 28°05′40″) of Hunan province, China, and named it A/environment/Hunan/3/2011(H5N1). Its genome consists of 8 single-stranded RNA segments, PB2, PB1, PA, HA, NP, NA, M, and NS, with 2,341, 2,341, 2,233, 1,776, 1,565, 1,399, 1,027, and 875 nucleotides, respectively (Table 1).

**TABLE 1:** Nucleotide sequence accession numbers

Influenza A virus segment [A/environment/Hunan/3/2011(H5N1)]	GenBank accession no.	Length of the segment (bp)
Segment 1	JX576794	2,341
Segment 2	JX576789	2,341
Segment 3	JX576791	2,233
Segment 4	JX576788	1,776
Segment 5	JX576792	1,565
Segment 6	JX576793	1,399
Segment 7	JX576787	1,027
Segment 8	JX576790	885

For this virus isolate, the protein encoded by the HA gene contains multiple basic amino acids adjacent to the cleavage site (RRRKR/G), which is considered a characteristic of highly pathogenic strains (4). The stalk of the NA protein has a 20-amino-acid deletion at site 49 to 68, which might be necessary for virus adaptation from wild birds to domestic fowl (chickens) (5). No amino acid substitutions related to possible enhancement of virus virulence to mammals, such as PB2-E627K or PB2-D701N (6, 7), have been found in the isolate. Its NS1 protein has Ala at 149, and a substitution from V to A at this site was reported to be related to virus replication in chickens (8).

The genome nucleotide sequence comparison showed that the HA gene of this isolate shares 99% homology with that of a Vietnam strain, A/Muscovy duck/Vietnam/LBM14/2011(H5N1), and other gene segments share high homology (≥95%) with east Asian H5N1 strains. Phylogenetic tree analysis indicates that the HA gene belongs to clade 2.3.2.1 and that other genes present different recombination features. These results suggest that the gene segments of this AIV H5N1 isolate have been under continuous evolution and recombination.

In conclusion, this study demonstrated that natural waters might play an important role in the transmission and perpetuation of the influenza virus. Strengthening epidemiology monitoring of influenza viruses is of important significance for understanding the mechanism of AIV transmission and will provide relevant information to prevent and control avian influenza.

### Nucleotide sequence accession numbers.

The genome sequences of A/environment/Hunan/3/2011(H5N1) have been deposited in GenBank under the accession numbers JX576787 to JX576794 (Table 1).
